# *catena*-Poly[[trimethyl­tin(IV)]-μ-5-methyl­thio­phene-2-carboxyl­ato-κ^2^ *O*:*O*′]

**DOI:** 10.1107/S1600536811049713

**Published:** 2011-11-25

**Authors:** Qi Zhu, Rufen Zhang

**Affiliations:** a College of Chemistry and Chemical Engineering, Liaocheng University, Shandong 252059, People’s Republic of China

## Abstract

In the title polymeric coordination compound, [Sn(CH_3_)_3_(C_6_H_5_O_2_S)]_*n*_, which contains two formula units in the asymmetric unit, the Sn^IV^ atom has a distorted trigonal–bipyramidal geometry, with two O atoms of the ligands in axial positions and three methyl groups in equatorial positions. Adjacent Sn^IV^ atoms are bridged by the ligands, thereby forming a chain propagating in [010].

## Related literature

For the biological activity of organotin compounds, see: Dubey & Roy (2003 ▶). For related structures, see: Wang *et al.* (2007) ▶; Ma *et al.* (2008 ▶). 
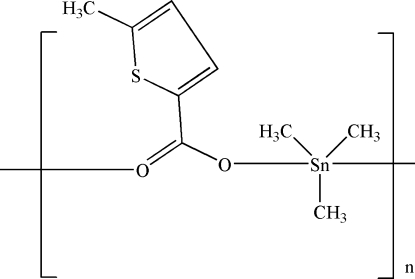


## Experimental

### 

#### Crystal data

[Sn(CH_3_)_3_(C_6_H_5_O_2_S)]
*M* *_r_* = 304.95
Triclinic, 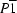

*a* = 9.9591 (8) Å
*b* = 10.0655 (11) Å
*c* = 13.9890 (12) Å
α = 69.385 (1)°
β = 72.225 (1)°
γ = 75.038 (2)°
*V* = 1231.9 (2) Å^3^
*Z* = 4
Mo *K*α radiation
μ = 2.21 mm^−1^
*T* = 298 K
0.44 × 0.15 × 0.09 mm

#### Data collection

Siemens SMART CCD diffractometer
Absorption correction: multi-scan (*SADABS*; Sheldrick, 1996 ▶) *T*_min_ = 0.443, *T*_max_ = 0.826
6219 measured reflections
4293 independent reflections
2340 reflections with *I* > 2σ(*I*)
*R*_int_ = 0.048

#### Refinement

*R*[*F* ^2^ > 2σ(*F* ^2^)] = 0.084
*wR*(*F* ^2^) = 0.242
*S* = 0.97
4293 reflections
243 parameters
H-atom parameters constrained
Δρ_max_ = 3.71 e Å^−3^
Δρ_min_ = −0.97 e Å^−3^

### 

Data collection: *SMART* (Siemens, 1996 ▶); cell refinement: *SAINT* (Siemens, 1996 ▶); data reduction: *SAINT*; program(s) used to solve structure: *SHELXS97* (Sheldrick, 2008 ▶); program(s) used to refine structure: *SHELXL97* (Sheldrick, 2008 ▶); molecular graphics: *SHELXTL* (Sheldrick, 2008 ▶); software used to prepare material for publication: *SHELXTL*.

## Supplementary Material

Crystal structure: contains datablock(s) I, global. DOI: 10.1107/S1600536811049713/hb6523sup1.cif

Structure factors: contains datablock(s) I. DOI: 10.1107/S1600536811049713/hb6523Isup2.hkl

Additional supplementary materials:  crystallographic information; 3D view; checkCIF report

## Figures and Tables

**Table 1:** Selected bond lengths (Å)

Sn1—C7	2.098 (12)
Sn1—C9	2.115 (14)
Sn1—C8	2.126 (16)
Sn1—O3	2.159 (9)
Sn1—O1	2.526 (10)
Sn2—C11	2.104 (14)
Sn2—C10	2.128 (16)
Sn2—C12	2.153 (15)
Sn2—O2	2.177 (10)
Sn2—O4^i^	2.502 (11)

Symmetry code: (i) 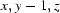.

**Fig. 1.**
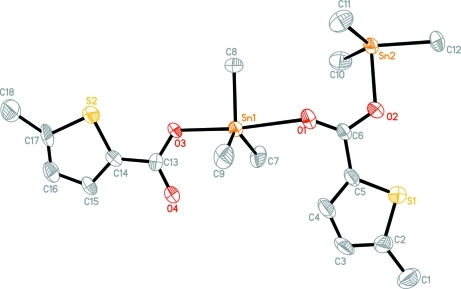
The asymmetric unit of the title compound, showing 30% probability displacement ellipsoids. H atoms have been omitted for clarity.

**Fig. 2.**
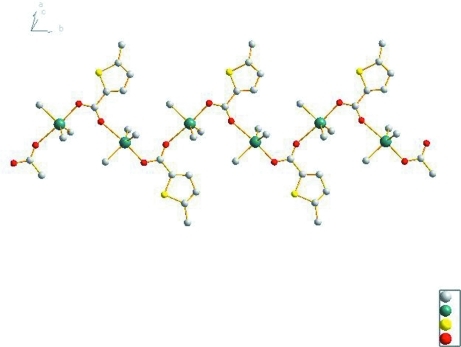
A view of the one-dimensional extended chain structure in the title compound.

## supplementary crystallographic information

### Comment

Organotin complexes are attracting more and more attention because of their considerable structural diversity and interesting topologies (Murugavel *et al.* 2001). Herein, we report the crystal structure of the title compound. The title compound,which is shown in Fig.2 forms an extended one-dimensional chain structure arising from Sn—O bridges formed by the 5-methyl-2-thiophenecarboxylic acid ligands. The Sn—O bond distances in the compound [Sn1—O1 = 2.526 (10) Å; Sn1—O3 = 2.159 (9) Å] are comparable to those found in a related organotin carboxylate (Ma *et al.*, 2008). The Sn atom is five-coordinate in a slightly distorted trigonal-bipyramidal coordination geometry, provided by the methyl groups in the equatorial positions and the two coordinated O atoms in the axial positions.

### Experimental

The reaction was carried out under a nitrogen atmosphere. 5-methyl-2-thiophenecarboxylic acid (1 mmol) and potassium hydroxide (1 mmol) were added to a stirred solution of methanol (30 ml) in a Schlenk flask and stirred for 0.5 h. trimethyltin chloride (1 mmol) was then added to the reactor and the reaction mixture was stirred for 12 h at room temperature. The resulting clear solution was evaporated under vacuum. The product was crystallized from a solution of diethyl ether to yield colorless blocks of the title compound (yield 76%). Anal. Calcd (%) for C_18_H_28_O_4_S_2_Sn_2_(Mr = 609.90):*C*, 35.44; H, 4.63. Found (%): C, 35.71; H, 4.39.

### Refinement

The H atoms were positioned geometrically, with methyl C—H distances of 0.96Å and aromatic C—H distances of 0.93 Å, and refined as riding on their parent atoms, with *U*_iso_(H) = 1.2 *U*_eq_(C) or 1.5 *U*_eq_(C) for the methyl groups.

### Crystal data

**Table:**

[Sn(CH_3_)_3_(C_6_H_5_O_2_S)]	*Z* = 4
*M**_r_* = 304.95	*F*(000) = 600
Triclinic, *P*1	*D*_x_ = 1.644 Mg m^−^^3^
*a* = 9.9591 (8) Å	Mo *K*α radiation, λ = 0.71073 Å
*b* = 10.0655 (11) Å	Cell parameters from 2063 reflections
*c* = 13.9890 (12) Å	θ = 2.8–26.3°
α = 69.385 (1)°	µ = 2.21 mm^−^^1^
β = 72.225 (1)°	*T* = 298 K
γ = 75.038 (2)°	Block, colorless
*V* = 1231.9 (2) Å^3^	0.44 × 0.15 × 0.09 mm

### Data collection

**Table:**

Siemens SMART CCD diffractometer	4293 independent reflections
Radiation source: fine-focus sealed tube	2340 reflections with *I* > 2σ(*I*)
graphite	*R*_int_ = 0.048
phi and ω scans	θ_max_ = 25.0°, θ_min_ = 2.6°
Absorption correction: multi-scan (*SADABS*; Sheldrick, 1996)	*h* = −11→11
*T*_min_ = 0.443, *T*_max_ = 0.826	*k* = −11→11
6219 measured reflections	*l* = −15→16

### Refinement

**Table:**

Refinement on *F*^2^	Primary atom site location: structure-invariant direct methods
Least-squares matrix: full	Secondary atom site location: difference Fourier map
*R*[*F*^2^ > 2σ(*F*^2^)] = 0.084	Hydrogen site location: inferred from neighbouring sites
*wR*(*F*^2^) = 0.242	H-atom parameters constrained
*S* = 0.97	*w* = 1/[σ^2^(*F*_o_^2^) + (0.1385*P*)^2^] where *P* = (*F*_o_^2^ + 2*F*_c_^2^)/3
4293 reflections	(Δ/σ)_max_ = 0.021
243 parameters	Δρ_max_ = 3.71 e Å^−^^3^
0 restraints	Δρ_min_ = −0.97 e Å^−^^3^

### Special details

**Table:**

Geometry. All e.s.d.'s (except the e.s.d. in the dihedral angle between two l.s. planes) are estimated using the full covariance matrix. The cell e.s.d.'s are taken into account individually in the estimation of e.s.d.'s in distances, angles and torsion angles; correlations between e.s.d.'s in cell parameters are only used when they are defined by crystal symmetry. An approximate (isotropic) treatment of cell e.s.d.'s is used for estimating e.s.d.'s involving l.s. planes.
Refinement. Refinement of *F*^2^ against ALL reflections. The weighted *R*-factor *wR* and goodness of fit *S* are based on *F*^2^, conventional *R*-factors *R* are based on *F*, with *F* set to zero for negative *F*^2^. The threshold expression of *F*^2^ > σ(*F*^2^) is used only for calculating *R*-factors(gt) *etc*. and is not relevant to the choice of reflections for refinement. *R*-factors based on *F*^2^ are statistically about twice as large as those based on *F*, and *R*- factors based on ALL data will be even larger.

### Fractional atomic coordinates and isotropic or equivalent isotropic displacement parameters (Å^2^)

**Table:**

	*x*	*y*	*z*	*U*_iso_*/*U*_eq_	
C18	−0.5657 (19)	1.292 (2)	0.007 (2)	0.118 (8)	
H18A	−0.6534	1.2537	0.0384	0.177*	
H18B	−0.5268	1.2810	−0.0618	0.177*	
H18C	−0.5841	1.3919	0.0029	0.177*	
C17	−0.4596 (16)	1.2104 (15)	0.0749 (16)	0.073 (5)	
Sn1	−0.05517 (11)	0.58071 (10)	0.21992 (7)	0.0541 (4)	
Sn2	0.05372 (12)	0.02160 (10)	0.27985 (8)	0.0585 (4)	
S1	0.3647 (5)	0.1708 (5)	0.4326 (4)	0.0753 (12)	
S2	−0.3972 (5)	1.0294 (5)	0.0923 (4)	0.0834 (14)	
O2	0.2015 (11)	0.1158 (10)	0.3134 (9)	0.070 (3)	
O3	−0.2091 (11)	0.7772 (10)	0.1936 (9)	0.073 (3)	
O1	0.1153 (12)	0.3438 (10)	0.2374 (9)	0.072 (3)	
C15	−0.3124 (18)	1.1534 (15)	0.1843 (14)	0.076 (5)	
H15	−0.2632	1.1724	0.2236	0.091*	
C2	0.4377 (16)	0.295 (2)	0.4463 (15)	0.075 (5)	
C6	0.1916 (14)	0.2509 (14)	0.2919 (11)	0.051 (3)	
C8	−0.172 (2)	0.4864 (19)	0.1640 (17)	0.099 (7)	
H8A	−0.1552	0.3837	0.1941	0.149*	
H8B	−0.1417	0.5104	0.0888	0.149*	
H8C	−0.2724	0.5226	0.1840	0.149*	
C14	−0.2966 (15)	1.0243 (15)	0.1721 (11)	0.055 (4)	
C13	−0.1981 (16)	0.8909 (16)	0.2101 (12)	0.060 (4)	
C7	−0.0941 (18)	0.5333 (18)	0.3835 (10)	0.075 (5)	
H7A	−0.1854	0.5857	0.4093	0.113*	
H7B	−0.0206	0.5606	0.4004	0.113*	
H7C	−0.0941	0.4319	0.4158	0.113*	
C5	0.2827 (15)	0.2953 (15)	0.3406 (12)	0.058 (4)	
C9	0.1179 (17)	0.6752 (18)	0.1082 (11)	0.081 (5)	
H9A	0.1680	0.7069	0.1426	0.122*	
H9B	0.0823	0.7560	0.0554	0.122*	
H9C	0.1820	0.6054	0.0758	0.122*	
C10	−0.1218 (18)	0.097 (2)	0.3910 (13)	0.089 (5)	
H10A	−0.1401	0.2000	0.3687	0.133*	
H10B	−0.0993	0.0624	0.4585	0.133*	
H10C	−0.2052	0.0612	0.3959	0.133*	
O4	−0.1108 (12)	0.8892 (11)	0.2585 (9)	0.078 (3)	
C11	0.0877 (18)	0.120 (2)	0.1165 (11)	0.083 (5)	
H11A	−0.0009	0.1408	0.0960	0.125*	
H11B	0.1566	0.0569	0.0806	0.125*	
H11C	0.1228	0.2085	0.0984	0.125*	
C12	0.175 (2)	−0.1893 (17)	0.334 (2)	0.125 (9)	
H12A	0.1702	−0.2493	0.2950	0.187*	
H12B	0.1350	−0.2312	0.4074	0.187*	
H12C	0.2724	−0.1816	0.3236	0.187*	
C3	0.398 (2)	0.4268 (19)	0.3810 (18)	0.095 (6)	
H3	0.4261	0.5099	0.3771	0.114*	
C4	0.3110 (19)	0.4263 (16)	0.3212 (14)	0.075 (5)	
H4	0.2759	0.5082	0.2726	0.090*	
C16	−0.4115 (16)	1.2583 (17)	0.1315 (14)	0.072 (5)	
H16	−0.4397	1.3516	0.1366	0.087*	
C1	0.5226 (19)	0.261 (2)	0.5266 (18)	0.114 (8)	
H1A	0.5870	0.1712	0.5275	0.171*	
H1B	0.4587	0.2526	0.5947	0.171*	
H1C	0.5763	0.3361	0.5090	0.171*	

### Atomic displacement parameters (Å^2^)

**Table:**

	*U*^11^	*U*^22^	*U*^33^	*U*^12^	*U*^13^	*U*^23^
C18	0.082 (13)	0.078 (14)	0.20 (2)	0.003 (11)	−0.076 (15)	−0.026 (15)
C17	0.056 (10)	0.031 (8)	0.103 (13)	0.008 (7)	−0.012 (9)	−0.003 (8)
Sn1	0.0729 (7)	0.0382 (6)	0.0567 (7)	−0.0075 (5)	−0.0288 (5)	−0.0104 (5)
Sn2	0.0853 (8)	0.0349 (6)	0.0607 (7)	−0.0059 (5)	−0.0363 (6)	−0.0077 (5)
S1	0.078 (3)	0.059 (3)	0.101 (3)	−0.011 (2)	−0.043 (2)	−0.021 (2)
S2	0.087 (3)	0.054 (3)	0.129 (4)	0.006 (2)	−0.059 (3)	−0.034 (3)
O2	0.086 (7)	0.041 (6)	0.098 (8)	−0.010 (5)	−0.046 (6)	−0.018 (5)
O3	0.094 (8)	0.031 (5)	0.117 (9)	0.003 (5)	−0.063 (7)	−0.026 (6)
O1	0.090 (8)	0.039 (6)	0.088 (8)	0.003 (5)	−0.035 (6)	−0.018 (5)
C15	0.098 (13)	0.034 (8)	0.105 (14)	0.001 (8)	−0.038 (11)	−0.026 (9)
C2	0.054 (9)	0.079 (13)	0.104 (14)	−0.001 (8)	−0.023 (9)	−0.047 (11)
C6	0.060 (9)	0.033 (8)	0.059 (9)	−0.006 (6)	−0.015 (7)	−0.013 (7)
C8	0.124 (16)	0.066 (12)	0.153 (19)	0.005 (11)	−0.096 (15)	−0.048 (12)
C14	0.062 (9)	0.043 (8)	0.062 (9)	−0.013 (7)	−0.009 (7)	−0.020 (7)
C13	0.065 (10)	0.048 (9)	0.064 (10)	−0.003 (7)	−0.027 (8)	−0.008 (7)
C7	0.103 (12)	0.074 (11)	0.036 (8)	0.005 (9)	−0.026 (8)	−0.007 (8)
C5	0.063 (9)	0.036 (8)	0.076 (10)	−0.004 (7)	−0.014 (8)	−0.023 (7)
C9	0.094 (12)	0.078 (12)	0.048 (9)	−0.027 (10)	−0.012 (9)	0.014 (8)
C10	0.104 (14)	0.106 (15)	0.068 (11)	−0.023 (11)	−0.015 (10)	−0.040 (11)
O4	0.098 (8)	0.040 (6)	0.109 (9)	−0.004 (5)	−0.056 (7)	−0.019 (6)
C11	0.093 (12)	0.106 (14)	0.045 (9)	−0.025 (11)	−0.019 (8)	−0.009 (9)
C12	0.17 (2)	0.028 (9)	0.21 (3)	0.010 (11)	−0.13 (2)	−0.020 (12)
C3	0.090 (13)	0.054 (11)	0.17 (2)	−0.002 (10)	−0.043 (14)	−0.065 (13)
C4	0.085 (12)	0.040 (9)	0.100 (13)	−0.005 (8)	−0.019 (10)	−0.028 (9)
C16	0.060 (10)	0.046 (9)	0.096 (13)	0.002 (8)	−0.005 (9)	−0.023 (9)
C1	0.074 (13)	0.137 (19)	0.17 (2)	0.014 (12)	−0.053 (14)	−0.091 (18)

### Geometric parameters (Å, °)

**Table:**

C18—C17	1.51 (2)	C8—H8B	0.9600
C18—H18A	0.9600	C8—H8C	0.9600
C18—H18B	0.9600	C14—C13	1.478 (19)
C18—H18C	0.9600	C13—O4	1.247 (17)
C17—C16	1.31 (2)	C7—H7A	0.9600
C17—S2	1.720 (15)	C7—H7B	0.9600
Sn1—C7	2.098 (12)	C7—H7C	0.9600
Sn1—C9	2.115 (14)	C5—C4	1.34 (2)
Sn1—C8	2.126 (16)	C9—H9A	0.9600
Sn1—O3	2.159 (9)	C9—H9B	0.9600
Sn1—O1	2.526 (10)	C9—H9C	0.9600
Sn2—C11	2.104 (14)	C10—H10A	0.9600
Sn2—C10	2.128 (16)	C10—H10B	0.9600
Sn2—C12	2.153 (15)	C10—H10C	0.9600
Sn2—O2	2.177 (10)	O4—Sn2^ii^	2.502 (11)
Sn2—O4^i^	2.502 (11)	C11—H11A	0.9600
S1—C2	1.690 (17)	C11—H11B	0.9600
S1—C5	1.694 (15)	C11—H11C	0.9600
S2—C14	1.694 (15)	C12—H12A	0.9600
O2—C6	1.270 (15)	C12—H12B	0.9600
O3—C13	1.281 (17)	C12—H12C	0.9600
O1—C6	1.232 (15)	C3—C4	1.37 (2)
C15—C14	1.333 (19)	C3—H3	0.9300
C15—C16	1.41 (2)	C4—H4	0.9300
C15—H15	0.9300	C16—H16	0.9300
C2—C3	1.36 (2)	C1—H1A	0.9600
C2—C1	1.50 (2)	C1—H1B	0.9600
C6—C5	1.51 (2)	C1—H1C	0.9600
C8—H8A	0.9600		
			
C17—C18—H18A	109.5	O4—C13—O3	121.8 (13)
C17—C18—H18B	109.5	O4—C13—C14	121.0 (14)
H18A—C18—H18B	109.5	O3—C13—C14	117.1 (13)
C17—C18—H18C	109.5	Sn1—C7—H7A	109.5
H18A—C18—H18C	109.5	Sn1—C7—H7B	109.5
H18B—C18—H18C	109.5	H7A—C7—H7B	109.5
C16—C17—C18	127.8 (16)	Sn1—C7—H7C	109.5
C16—C17—S2	111.3 (13)	H7A—C7—H7C	109.5
C18—C17—S2	120.7 (16)	H7B—C7—H7C	109.5
C7—Sn1—C9	125.7 (7)	C4—C5—C6	128.5 (15)
C7—Sn1—C8	116.7 (8)	C4—C5—S1	111.4 (13)
C9—Sn1—C8	116.3 (8)	C6—C5—S1	120.1 (10)
C7—Sn1—O3	97.3 (5)	Sn1—C9—H9A	109.5
C9—Sn1—O3	94.5 (6)	Sn1—C9—H9B	109.5
C8—Sn1—O3	89.4 (5)	H9A—C9—H9B	109.5
C7—Sn1—O1	87.2 (5)	Sn1—C9—H9C	109.5
C9—Sn1—O1	86.3 (5)	H9A—C9—H9C	109.5
C8—Sn1—O1	84.9 (5)	H9B—C9—H9C	109.5
O3—Sn1—O1	173.9 (4)	Sn2—C10—H10A	109.5
C11—Sn2—C10	123.3 (7)	Sn2—C10—H10B	109.5
C11—Sn2—C12	117.6 (9)	H10A—C10—H10B	109.5
C10—Sn2—C12	117.7 (9)	Sn2—C10—H10C	109.5
C11—Sn2—O2	99.4 (6)	H10A—C10—H10C	109.5
C10—Sn2—O2	92.2 (6)	H10B—C10—H10C	109.5
C12—Sn2—O2	89.8 (6)	C13—O4—Sn2^ii^	147.4 (10)
C11—Sn2—O4^i^	86.2 (6)	Sn2—C11—H11A	109.5
C10—Sn2—O4^i^	87.3 (6)	Sn2—C11—H11B	109.5
C12—Sn2—O4^i^	84.7 (6)	H11A—C11—H11B	109.5
O2—Sn2—O4^i^	173.5 (3)	Sn2—C11—H11C	109.5
C2—S1—C5	92.3 (8)	H11A—C11—H11C	109.5
C14—S2—C17	91.5 (9)	H11B—C11—H11C	109.5
C6—O2—Sn2	122.2 (9)	Sn2—C12—H12A	109.5
C13—O3—Sn1	123.0 (9)	Sn2—C12—H12B	109.5
C6—O1—Sn1	145.3 (9)	H12A—C12—H12B	109.5
C14—C15—C16	113.1 (16)	Sn2—C12—H12C	109.5
C14—C15—H15	123.5	H12A—C12—H12C	109.5
C16—C15—H15	123.5	H12B—C12—H12C	109.5
C3—C2—C1	127.4 (18)	C2—C3—C4	114.1 (16)
C3—C2—S1	109.6 (13)	C2—C3—H3	122.9
C1—C2—S1	122.8 (16)	C4—C3—H3	122.9
O1—C6—O2	125.7 (13)	C5—C4—C3	112.5 (17)
O1—C6—C5	119.8 (12)	C5—C4—H4	123.7
O2—C6—C5	114.5 (12)	C3—C4—H4	123.7
Sn1—C8—H8A	109.5	C17—C16—C15	112.9 (15)
Sn1—C8—H8B	109.5	C17—C16—H16	123.5
H8A—C8—H8B	109.5	C15—C16—H16	123.5
Sn1—C8—H8C	109.5	C2—C1—H1A	109.5
H8A—C8—H8C	109.5	C2—C1—H1B	109.5
H8B—C8—H8C	109.5	H1A—C1—H1B	109.5
C15—C14—C13	128.9 (15)	C2—C1—H1C	109.5
C15—C14—S2	111.0 (12)	H1A—C1—H1C	109.5
C13—C14—S2	119.8 (10)	H1B—C1—H1C	109.5
			
C16—C17—S2—C14	−3.4 (14)	C17—S2—C14—C13	−174.2 (12)
C18—C17—S2—C14	−179.0 (16)	Sn1—O3—C13—O4	−12 (2)
C11—Sn2—O2—C6	58.4 (13)	Sn1—O3—C13—C14	169.9 (9)
C10—Sn2—O2—C6	−65.9 (12)	C15—C14—C13—O4	−2(3)
C12—Sn2—O2—C6	176.4 (13)	S2—C14—C13—O4	171.4 (13)
O4^i^—Sn2—O2—C6	−152 (3)	C15—C14—C13—O3	175.9 (16)
C7—Sn1—O3—C13	65.4 (13)	S2—C14—C13—O3	−10.4 (19)
C9—Sn1—O3—C13	−61.4 (13)	O1—C6—C5—C4	−11 (2)
C8—Sn1—O3—C13	−177.8 (14)	O2—C6—C5—C4	169.6 (15)
O1—Sn1—O3—C13	−158 (3)	O1—C6—C5—S1	169.1 (11)
C7—Sn1—O1—C6	−23.1 (18)	O2—C6—C5—S1	−10.3 (17)
C9—Sn1—O1—C6	103.0 (18)	C2—S1—C5—C4	0.3 (13)
C8—Sn1—O1—C6	−140.2 (19)	C2—S1—C5—C6	−179.8 (12)
O3—Sn1—O1—C6	−160 (3)	O3—C13—O4—Sn2^ii^	146.7 (14)
C5—S1—C2—C3	0.1 (14)	C14—C13—O4—Sn2^ii^	−35 (3)
C5—S1—C2—C1	175.3 (15)	C1—C2—C3—C4	−175.3 (18)
Sn1—O1—C6—O2	136.9 (14)	S1—C2—C3—C4	0(2)
Sn1—O1—C6—C5	−42 (2)	C6—C5—C4—C3	179.5 (15)
Sn2—O2—C6—O1	−11 (2)	S1—C5—C4—C3	−0.5 (19)
Sn2—O2—C6—C5	167.9 (9)	C2—C3—C4—C5	1(2)
C16—C15—C14—C13	176.4 (14)	C18—C17—C16—C15	−179.6 (18)
C16—C15—C14—S2	2.2 (19)	S2—C17—C16—C15	5(2)
C17—S2—C14—C15	0.6 (14)	C14—C15—C16—C17	−5(2)

Symmetry codes: (i) *x*, *y*−1, *z*; (ii) *x*, *y*+1, *z*.
